# P-215. Screening to Prophylax against *Clostridioides difficile* Infection: A Trial of Primary Prophylaxis in *C. difficile* Carriers

**DOI:** 10.1093/ofid/ofae631.419

**Published:** 2025-01-29

**Authors:** Carmen DeMarco, David Sengstock, Elie Mulhem, Christopher F Carpenter, Paul Chittick, Paul Johnson, Angela M Saputo, Marithel Sandoval-Yemmans, Maureen Cooney, Matthew Sims

**Affiliations:** Corewell Health East - William Beaumont University, Royal Oak, Michigan; Wayne State University, Canton, Michigan; Corewell Health, Sterling Heights, Michigan; Oakland University William Beaumont School of Medicine, Royal Oak, Michigan; Corewell Health, Sterling Heights, Michigan; Corewell Health William Beaumont University Hospital, Royal Oak, Michigan; Corewell Health, Sterling Heights, Michigan; Corewell Health Research Institute, Royal Oak, Michigan; Corewell Health, Sterling Heights, Michigan; William Beaumont University Hospital, Corewell Health, Royal Oak, Michigan

## Abstract

**Background:**

*Clostridioides difficile* infection (CDI) is one of the five urgent threats identified by the Centers for Diseases Control and Prevention (CDC). In addition to actual infection, *C. difficile* can colonize patients, particularly those with other gastrointestinal pathologies and with frequent healthcare exposure. Several studies have shown the potential benefit of secondary prophylaxis to prevent recurrence of CDI in patients with a recent prior infection. The Screening to Prophylax against CDI study (StoP CDI) was a double blinded randomized placebo-controlled trial of vancomycin primary prophylaxis for CDI in patients on high-risk antibiotics who were asymptomatic carriers of *C. difficile*. The study also aimed to determine the carriage rate of *C. difficile* in the hospitalized population and whether carriage of *C. difficile* was a risk factor for CDI.

**Methods:**

All adult inpatients in three Detroit-area hospitals were screened for new antibiotic starts and then for inclusion and exclusion criteria. Patients who qualified were consented to be screened by PCR for the presence of toxigenic *C. difficile,* and if positive were approached to participate in the trial of prophylaxis. Carriage rates in all screened patients were determined as were rates of CDI in the groups receiving vancomycin prophylaxis or placebo, those who were carriers but declined to participate in the randomized portion of the study, and those who were not carriers.

**Results:**

1294 patients consented to be screened and 728 provided a stool sample, of whom 81 (11.1%) were found to be carriers of toxigenic *C. difficile*. Twenty-seven patients agreed to be randomized, 13 were randomized to vancomycin prophylaxis and 14 to placebo. None of the randomized patients developed CDI; however, 2 of 48 evaluable carriers who were not randomized developed CDI, corresponding to a rate of CDI in non-prophylaxed carriers of 4.2%. Of 640 evaluable non-carriers, 4 were diagnosed with CDI giving a rate of 0.63%.

**Conclusion:**

Over 10% of adult hospitalized patients are carriers of *C. difficile*. The risk of developing CDI among carriers was 6.7x higher than in non-carriers. Vancomycin prophylaxis in carriers may reduce the risk for CDI but the number of patients was too small to fully test this hypothesis.

**Disclosures:**

**Christopher F. Carpenter, MD**, AstraZeneca: Grant/Research Support|Cidara Therapeutics: Advisor/Consultant|Dompe Farmaceutici: Grant/Research Support|F2G LTD: Advisor/Consultant|Rebiotix: Advisor/Consultant **Matthew Sims, MD, PhD**, Adaptive Phage Therapeutics: Principal Investigator Clinical Trial|Applied Biocode: Advisor/Consultant|Applied Biocode: Principal Investigator Clinical Trial|Astra Zeneca: Sub-investigator Clinical Trial|Biotest AG: Principal Investigator Clinical Trial|ContraFect: Principal Investigator Clinical Trial|Dompe: Sub-investigator Clinical Trial|Janssen: Principal Investigator Clinical Trial|Leonard-Meron Biosciences: Principal Investigator Clinical Trial|Novozyme: Principal Investigator Clinical Trial|OpGen: Advisor/Consultant|OpGen: Principal Investigator Clinical Trial|Pfizer: Principal Investigator Clinical Trial|Prenosis: Advisor/Consultant|Prenosis: Principal Investigator Clinical Trial|QIAGEN: Principal Investigator Clinical Trial|Seres: Advisor/Consultant|Seres: Principal Investigator Clinical Trial|Venatorx: Advisor/Consultant

